# Metagenomic Exploration of *Atelerix albiventris* Gut Microbiome

**DOI:** 10.1128/MRA.01342-20

**Published:** 2021-01-07

**Authors:** Makhabbat Amanbayeva, Elmira Anarkulova, Andrey Bogoyavlenskiy, Madina Alexyuk, Anar Imangazy, Vladimir Berezin

**Affiliations:** a Abai Kazakh National Pedagogical University, Almaty, Kazakhstan; b Research and Production Center for Microbiology and Virology, Almaty, Kazakhstan; DOE Joint Genome Institute

## Abstract

Here, we report the metagenomic analysis of the gut of *Atelerix albiventris*, an animal typically kept as a pet in Kazakhstan. In this case, shotgun metagenomic sequencing of the RNA and DNA viral community was performed.

## ANNOUNCEMENT

Nowadays, pets are becoming more and more important (1, 2). At the same time, not only familiar cats and dogs but also exotic hedgehogs, lizards, snails, and spiders are being acquired as pets. The introduction of exotic domestic pets in the house creates the need for a more thorough study of the pets’ ability to transmit infectious diseases to humans; therefore, the investigation of their microbiomes is an important task in assessing the epidemiological well-being of the population.

Here, the viral and microbial community from the feces of a healthy white-bellied pygmy hedgehog, *Atelerix albiventris* (an ordinary representative of pet shops and minizoos in Almaty, Kazakhstan [43°13′38.6″N, 76°51′52.2″E]), is presented.

Two grams of feces was homogenized in 18 ml of phosphate-buffered saline using the IKA Ultra-Turrax disposable workstation with a disperser tube, filtered using a 0.45-μm membrane to remove most bacteria, and concentrated by ultracentrifugation using a Beckman Coulter Avanti J30I ultracentrifuge at 29,000 rpm for 2 h at 4°C.

Viral RNA was extracted using a QIAamp viral RNA minikit (Qiagen). The sample extracts were pretreated with RNase-free DNase (Promega). Double-stranded cDNA was obtained with a SuperScript double-stranded cDNA synthesis kit (Invitrogen) according to the manufacturer’s instructions. Total DNA was isolated using a PureLink genomic DNA extraction kit (Thermo Fisher Scientific, USA) and stored at −80°C. Genomic DNA and synthesized double-stranded cDNA were pooled. DNA libraries were prepared from 1 ng of the pooled isolated nucleic acids using the Nextera XT DNA sample preparation kit (Illumina, USA). High-throughput sequencing was performed by using an Illumina MiSeq system (paired-end sequencing [2 × 300 bp] with a MiSeq kit v3).

The resulting sequences (852,082 reads) were tested for quality using FastQC v0.11.9 (https://www.bioinformatics.babraham.ac.uk/projects/fastqc) and Trimmomatic v0.36 (3) from the Genome Detective tool (4). After the removal of low-quality reads (Q scores of <30) and adapter trimming, 766,874 reads were analyzed by the Kaiju program (5). All tools were run with default parameters unless otherwise specified.

**FIG 1:**
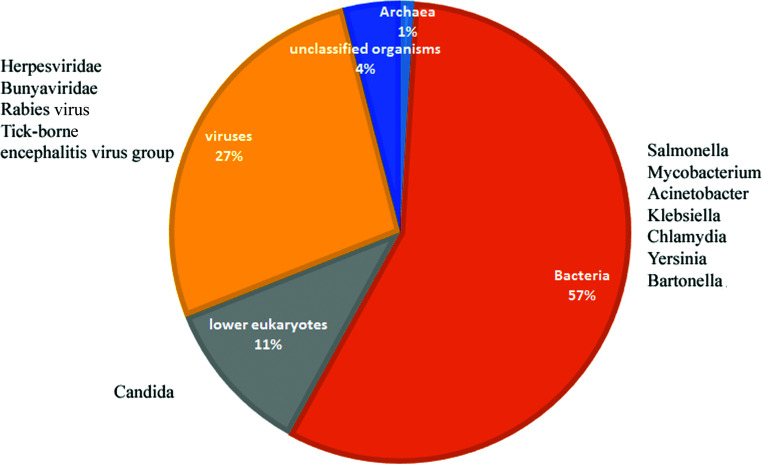
Taxonomic diversity of the microbial and viral communities of *Atelerix albiventris*. Taxonomic assignment of the metagenomic reads was based on the NCBI BLAST nonredundant plus eukaryotes database using Kaiju.

Taxonomic classification of metagenomic data for the *Atelerix albiventris* feces showed that 1% of the sequences corresponded to archaea, 11% to lower eukaryotes, 27% to viruses, 57% to bacteria, and 4% to unclassified organisms (Fig. 1). Although most of the sequences belonged to either normal microflora or microorganisms from food, unfortunately, 1 to 2% of the reads were assigned to microflora potentially capable of causing a number of diseases in humans, including sequences of representatives of *Candida*, *Salmonella*, *Mycobacterium*, Acinetobacter, *Klebsiella*, *Chlamydia*, *Yersinia*, *Bartonella*, *Herpesviridae*, *Bunyaviridae*, rabies virus, and the tick-borne encephalitis virus group.

### Data availability.

Raw sequence reads are available under BioProject accession number PRJNA656141 and SRA accession number SRR12422733.
